# Adherence to a Mediterranean diet in Morocco and its correlates: cross-sectional analysis of a sample of the adult Moroccan population

**DOI:** 10.1186/1471-2458-12-345

**Published:** 2012-05-11

**Authors:** Karima El Rhazi, Chakib Nejjari, Dora Romaguera, Catherine Feart, Majdouline Obtel, Ahmed Zidouh, Rachid Bekkali, Pascale Barberger Gateau

**Affiliations:** 1 Department of epidemiology and Public Health, Faculty of Medicine, Fez, 30000, Morocco; 2 Department of Epidemiology and Biostatistics, School of Public Health, Imperial College London, London, W2 1PG, UK; 3 Inserm, U897, Bordeaux, F-33076, France; 4 Université Bordeaux Segalen, Bordeaux, F-33076, France; 5 Association Lalla Salma de Lutte Contre le cancer, Rabat, 10000, Morocco

## Abstract

**Background:**

Dietary habits in Morocco are changing and the causes are not well understood. This study aimed to analyse socio-demographic factors associated with adherence to the Mediterranean diet (MeDi) in a national random sample of the adult Moroccan population.

**Methods:**

The data collected in this cross-sectional survey included socio-demographic factors and a food frequency questionnaire. MeDi adherence was assessed in 2214 individuals with complete dietary data. MeDi adherence was measured according to a simplified MeDi score based on the weekly frequency of intake of eight food groups (vegetables, legumes, fruits, cereal or potatoes, fish, red meat, dairy products and olive oil) with the use of the sex specific medians of the sample as cut-offs. A value of 0 or 1 was assigned to consumption of each component according to its presumed detrimental or beneficial effect on health. Logistic regression was used to estimate the association between MeDi adherence (low score 1-4 vs. high 5-8) and other factors.

**Results:**

Mean age of the sample was 41.4 (standard deviation 15.3) years, 45.4% were men and 29.9% had a low MeDi adherence. Married subjects (adjusted odds ratio ORa=0.68, 95% CI 0.55-0.84) were less likely to have a low MeDi adherence compared to single, divorced or widowed persons. Persons from rural areas (ORa=1.46, 95% CI: 1.02-2.08), were more often low MeDi adherents compared to those from urban areas. Obese persons (ORa=1.56, 95% CI: 1.16-2.11) were more prone to low MeDi adherence than normal weight individuals.

**Conclusion:**

MeDi is far from being a universal pattern in the Moroccan population. Intervention strategies should be implemented in target groups to maintain the traditional MeDi pattern considered as the original diet in Morocco.

## Background

The Mediterranean Diet (MeDi) is the dietary pattern usually consumed among the populations bordering the Mediterranean Sea. Many studies consider the MeDi as a model of healthy eating and have reported its contribution to a favorable health status, a reduced risk of many chronic diseases and a better quality of life [1-6].

Common components of the traditionally defined MeDi are: abundant intake of plant foods (fruits, vegetables, breads, cereals, beans, nut and seeds); olive oil as the principal source of added fat; moderate consumption of wine; low to moderate consumption of cheese, yoghurt, fish, poultry and eggs; and low consumption of red and processed meat [1,7].

There is no single MeDi pattern. The dietary practices of countries bordering the Mediterranean Sea vary considerably; even within the same country, significant differences in dietary patterns exist [8]. Furthermore, many studies have showed great intake variations among Mediterranean countries and concluded that there is a Westernization of dietary habits in the Mediterranean region [9-11].

Morocco is located on the south west coast of the Mediterranean Sea. This country is undergoing a demographic and epidemiological transition. The proportion of the Moroccan population living in urban areas increased from 29.0% in 1960 to 55.1% in 2004 [12]. Life expectancy at birth increased from 47.0 years in 1962 to 72.2 years in 2007 [13]. In parallel, dietary habits, that were supposed to follow a Mediterranean pattern, have changed considerably [14,15]. During last decades, the consumption of red meat increased and was accompanied by a steadily increasing consumption of bread [15]. The causes of this nutritional transition are not well understood and it is important for policy makers to have accurate information not only about dietary shifting but also about socioeconomic and demographic factors that are related to dietary habits in Morocco.

### Objective

The present study aimed to identify demographic, socio-economic and lifestyle factors associated with adherence to a Mediterranean diet in a sample of adult Moroccan population.

## Methods

The present study is a population-based cross-sectional survey carried out in May 2008.

### Sampling

The target population consisted of all Moroccan subjects aged 18 years and above. The theoretical sample size was set at 3000 individuals in order to provide a precision of 2%, for a 15% risk factor prevalence, 95% confidence interval and a cluster effect of 2 taking into account an anticipated 80% participation rate. The sampling technique included stratification according to geographical area (urban, rural). A cluster, which was a neighborhood in urban area and locality in rural area, of 20 households was selected at random from each of the 150 previously randomized and size proportionally selected communes. One person (men and women alternatively) aged 18 years or above was selected at random from each household of the cluster to take part in the survey. Exclusion criteria included subjects seeking medical attention due to a severe acute illness.

This study was conducted according to the guidelines laid down in the Declaration of Helsinki. It was approved by the ethics committee of Fez University Hospital Center. All subjects gave their consent before answering the survey.

### Questionnaire

The questionnaire was divided into two parts. The first part included questions on demographic factors (age, sex, origin area), socio-economic status (education, monthly family income, occupation, housing category) and life style (tobacco consumption and physical activity which included usual physical activity at work, for going to and from work, and in leisure-time). The second part of the questionnaire comprised a food frequency questionnaire (FFQ) that included 38 foods and beverages commonly consumed in Morocco. Actually, ten food groups were considered (bread/cereals, rice/semolina, vegetables, legumes, fresh fruits, dairy products including milk, yogurt and cheese, red meat including veal, mutton, lamb, camel, goat…, poultry, fish, eggs), six types of beverages (milk, coffee, green and black tea, juice and soda) and five types of added fats (olive oil, argan oil, other vegetable oil, butter, cream or mayonnaise). For each participant, frequency of intake of each of the indicated food groups was reported in usual number of days of consumption per week. Eight frequency categories were proposed (less than one day/week, 1 day/week, 2/week, 3/week, 4/week, 5/week, 6/week, 7/week) for each food item and beverage except for oil. For fresh fruits, both weekly frequency of intake (number of days of consumption/week) and number of portions on a typical day of intake were reported. Portion size was not assessed. For each fat type, two frequency categories were available: regular consumer (fat consumed either for dressing or cooking) vs. non consumer.

The data were collected in the subjects’ homes during a personal interview carried by a trained pair (one man and one woman) including physicians and nurses. The questionnaire was administered homogeneously from Monday to Sunday. Its face validity was examined in a pilot study in 20 participants and showed that the FFQ was acceptable and understandable.

### Variables studied

#### A simplified Mediterranean-diet score

A simplified score of MeDi adherence was constructed following an adaptation of previously described MeDi scores [16,17] to take into account the available data. In the present study, computation of the simplified MeDi adherence score was based on the self-reported frequency of weekly intake of each food group except for fruit where the total weekly intake was calculated by multiplying the number of days of intake per week by the number of portions consumed daily. Several details about red meat consumption were reported. The frequency of consumption of high temperature cooked red meat, grilled or roasted red meat, traditional processed red meat (Khliaa and Qaddid) and modern processed red meat were added to compute the total frequency of consumption for the “red meat group” in the simplified MeDi score. For cereals, frequency of consumption was computed by adding the frequencies of the bread/crackers/cereal item and rice/pasta/semolina items. A value of 0 or 1 was assigned to each of these eight components (bread/cereals/riz/semolina, vegetables, fresh fruits, legumes, dairy products, red meat, fish, fat intake) using the sex-specific medians of the sample as cut-off. For components that were expected to be beneficial to health (vegetables, legumes, fresh fruits, cereals and fish), persons whose consumption was below the median were assigned a value of 0, and persons whose consumption was at or above the median were assigned a value of 1. For components presumed to be detrimental for health (red meat and dairy products), persons whose consumption was below the median were assigned a value of 1, vs. 0 for the others. For fat intake, as we could not compute the mono-unsaturated fatty acid (MUFA) to saturated fatty acid ratio [16,17], we used a proxy for the computation of the simplified MeDi score. Indeed, we considered olive oil intake as the main dietary source of MUFA in Morocco in the absence of pork consumption for religious reasons. Hence, persons who consumed olive oil for dressing or for cooking were assigned a value of 1, and 0 for non-consumers. A moderate consumption of alcohol is usually considered as a component of the traditional MeDi [16]. Given that alcohol drinking is forbidden for religious reasons, and therefore not usual or probably underreported in Moroccan population, this component was not considered for the simplified MeDi score computation. Thus, the total simplified MeDi score ranged from 0 (minimal adherence to the traditional MeDi) to 8 (maximal adherence). Adherence to the MeDi was considered dichotomously: a simplified MeDi score ranging from 0 to 4 represented a low MeDi adherence whereas a simplified MeDi score ranging from 5 to 8 a high MeDi adherence. The score categories were defined so as to be close to the first tertile of the distribution of the diet score in our sample. Indeed, we made the assumption that Moroccan population still grossly adheres to MeDi and we wanted to identify people at high risk to depart from this type of diet.

#### Other variables

We examined monthly income of household, housing, occupation, education, marital status, gender, and place of residence (urban or rural). Income was recorded as a class variable with the following categories: < 2000 MAD which is the minimal salary in Morocco, 2000 to 4999 MAD, and 5000 ≥ MAD or greater (the equivalent currency exchange is: 1 MAD = 0.09 Euro). Occupation was grouped into two classes: active or student, vs. retired or unemployed or housewife, the latter being used only in women. For statistical analyses, retired and unemployed individuals were grouped with housewives due to their small number and because of the difficulty to compare housewives which were all women, with other categories of occupation. Housing was classified according to criteria used previously (Moroccan Health Ministry, 2003) into four categories: luxurious or modern, new medina, old medina, poor housing or slums. Level of education was recorded into three categories: university or secondary level (≥ 6 years of schooling), primary level or informal education or coranic school ([1–6 years[), and illiterates (less than one year of schooling). Marital status was grouped into two classes: married vs single or divorced or widowed. Age was grouped into three categories (< 35; 35 – 54 and ≥ 55 years) based on the tertile distribution of our sample.

Concerning anthropometric variables, body weight and height were measured, and body mass index (BMI) was computed as the person’s weight divided by the person’s size squared (kg/m2). BMI was used to define overweight (BMI ≥ 25 and < 30 kg/m2) and obesity (BMI ≥ 30 kg/m2) according to WHO recommendations [18].

Concerning tobacco consumption, respondents were classified as current smokers (daily and occasional smokers) if they were smoking at the time of the survey; they were classified as ex-smokers if they stopped smoking during more than 3 months at the time of the survey; and they were classified as never smokers if they had never smoked in their lifetime.

Physical activity was assessed through a questionnaire which included items about moderate and vigorous activities that are performed at work and during leisure time in a usual week of the previous month. Data about transportation either by walking or cycling were also collected. For each item, number of days per week and number of minutes per day were reported. Physical activity was then defined according to WHO guidelines which recommend practicing at least 30 minutes of regular, moderate or intensive physical activity on most days [19].A person was therefore considered as active if he/she is performed at least 30 minutes of physical activity (including household activities) per day.

#### Statistical analysis

Food frequency consumption was described in the whole population study and in men and women separately. Sociodemographic characteristics of the sample were described according to high (simplified MeDi score 5–8) vs low (simplified MeDi score 0–4) MeDi adherence. Student’s *t*-test was used for comparison of means and the chi-square tests for comparison of proportions between the two groups. Factors associated with p value < 0.20 in univariate analyses were included in the multivariate logistic model. A Multivariate logistic regression analysis was used to estimate adjusted odds ratios and 95% confidence intervals (CI) in subjects without any missing data in one or several covariates (N = 2183). The level of significance was established at p < 0.05. Statistical analyses were carried out using Statistical Package for the Social Sciences (SPSS) v17.

## Results

### Characteristics of the population study

In total, out of the 3000 people selected, 2891 (96.5%) took part in the survey, including 1430 men (49.5%) and 1461 women (50.5%). People who are not included in the survey had refused to participate or were absent (3.5%). Among the 2891, individuals with at least one missing data in at least one component of simplified MeDi adherence score (N = 677, 23.4% of the sample) were excluded from the computation of the score. The present study is thus based on 2214 individuals with complete dietary data. More missing data were observed in males than in females (29.8% vs 17.4%; p < 0.001), in those of urban origin than in rural (25.0% vs 21.6%; p = 0.03). But there was no statistical difference in mean age between subjects with complete data (41.39 years, SD 15.31) vs subjects with missing data (42.15 years, SD14.97); p = 0.25.

The sample description according to sex is shown in Table 1. Among the 2214 who have answered all FFQ items, 45.4% were men and 42.8% were from rural areas. Mean age was 41.4 (standard deviation, SD 15.3, median 40.0) years. Regarding education, 56.8% of women were illiterate vs 30.1% of men (p < 0.0001). The proportion of participants owning less than 2000 MAD/month seemed to be higher in men but 23.2% of the women did not know the amount of family income. For housing, 52.0% of the sample was living in slums or poor housing.

**Table 1:** Main demographic and socioeconomic characteristics according to gender

	***All population***	***Women***	***Men***	***p***
***Place of residence***				
***N***	2214	1208	1006	
Rural area	42.8	40.8	45.2	0.03
Urban area	57.2	59.2	54.8	
***Age groups (years)***				
***N***	2212	1206	1006	
< 35	38.1	43.0	32.3	<0.0001
35 - 49	32.5	34.7	29.8	
≥ 50	29.4	22.3	37.9	
***Marital status***				
***N***	2193	1194	999	
Single/Divorced/widowed	31.5	34.8	27.5	<0.0001
Married	68.5	65.2	72.5	
***Education***				
***N***	2204	1203	1001	
Illiterate	44.6	56.8	30.0	<0.0001
< 6 years	29.2	21.9	37.8	
≥ 6 years	26.2	21.3	32.2	
***Occupation***				
***N***	2186	1186	1000	
Active/student	45.7	18.0	78.5	<0.0001
Retired/unemployed/Housewife	54.3	82.0	21.5	
***Average family income (MAD)***				
***N***	2108	1123	985	
< 2000	52.2	44.6	60.9	<0.0001
2 000 à 4 999	25.7	24.2	27.4	
≥ 5 000	08.9	08.0	09.9	
Don’t know	13.2	23.2	01.8	
***Housing***				
***N***	2214	1208	1006	
Luxurious or modern	13.6	15.3	11.4	0.04
New medina	15.4	15.5	15.2	
Old medina	19.0	18.0	20.5	
Poor housing or slums	52.0	51.2	52.9	
***Tobacco consumption***				<0.001
Current smoker	15.9	0.7	30.1	
Ex-smoker	9.1	0.5	17.1	
Never smoker	75.1	98.7	52.8	
***Physical activity > = 30 min/day***				
***N***	2032	1134	898	
Yes	61.6	59.1	63.6	00.040
***BMI class (Kg/m2)***				
***N***	2204	1201	1003	
< 25	56.3	47.4	67.0	<0.0001
25 – 29.9	30.1	32.8	26.8	
≥ 30	13.6	19.8	6.2	

Sample of the adult Moroccan population, 2008.

The proportion of persons considered as physically active was similar in both genders (63.6% and 59.1% in men and women respectively). The prevalence of obesity (BMI ≥ 30 Kg/m2) in the whole sample was 13.6% but obesity was much more frequent in women than in men (19.8% vs 6.2%; p < 0.0001).

### Food intake

The consumption of food groups by gender is described in Table 2. Medians of food group consumption were similar in men and women except for dairy products which were more frequently consumed by women. The daily food pattern consisted of cereals and vegetables. Fresh fruits were consumed about three days/week. Red meat was consumed twice a week in median. About one half of the sample consumed fish and legume once a week. Olive oil and other vegetable oils were the main sources of fat either for cooking or dressing.

**Table 2:** Food consumption according to MeDi† adherence among a sample of the adult Moroccan population, 2008

**MeDi† adherence**	**Low (1–4)N = 662**		**High (5–8)N = 1552**	
	**Male**	**Female**	**Male**	**Female**
Food categories	m (SD)	m (SD)	m (SD)	m (SD)
Bread/rice/potatoes/pasta*	9.6 (2.2)	9.5 (2.3)	11.0 (2.3)	10.9 (2.4)
Dairy products*	3.8(4.7)	6.4(5.6)	4.0 (4.6)	5.6 (5.7)
Vegetables*	5.3(2.0)	6.4 (1.5)	6.2 (1.6)	6.8 (0.7)
Legumes*	0.9 (1.2)	0.6 (1.0)	1.6 (1.1)	1.3 (1.1)
Red meat*	02.9 (2.6)	3.8 (3.1)	2.5 (2.7)	2.8 (3.0)
Fish*	1.2 (1.6)	1.1 (1.5)	1.5 (1.3)	1.6 (1.6)
Fruits**	4.1 (6.5)	5.0 (6.5)	5.4 (5.9)	6.3 (6.3)
Olive oil (%)	70.0	59.7	89.3	86.9

All P values <0.05 for *t*-test for Equality of means or for the Chi2 test according to gender among categories of simplified MeDi score.

*Number of days of usual consumption per week, **Number of servings per week.

† Mediterranean Diet.

### Simplified **Mediterranean diet adherence score**

The simplified MeDi score ranged from 1 to 8, with 31 persons in the extreme values [1,8]; 29.9% had a simplified MeDi score lower than 5. The mean simplified MeDi score was 5.1 (SD 1.2).

As expected, greater MeDi adherence was characterized by higher intake of vegetables, fresh fruits, legumes, cereals, fish and olive oil and by lower intake of red meat and dairy products (Table 2).

The associations between demographic and socio economic factors and MeDi adherence are shown in Table 3. There was no significant association between MeDi adherence and age, gender, education, income, occupation and physical activity. However, low MeDi adherence was associated with being single, divorced or widowed. It was also and surprisingly strongly associated with rural living (p = 0.002). According to housing category, new and old medina residents were more likely to follow a MeDi than residents in luxurious or modern housing or those living in poor housing. A post-hoc pairwise test showed a significant difference between old medina and both luxurious or modern housing and poor housing or slums. The association with BMI was of borderline significance, with obese individuals tending to have lower MeDi adherence.

**Table 3:** Characteristics by categories of MeDi adherence

		**Simplified MeDi† score category**		
	**N**	**Low (1–4)**	**High (5–8)**	**p**
***Place of residence***				0.002
Urban	1266	27.3	72.7	
Rural	948	33.3	66.7	
***Gender***				0.13
Women	1208	28.6	71.4	
Men	1006	31.5	68.5	
***Age group***				0.80
< 35 years	843	30.4	69.6	
35 – 49 years	718	29.5	70.5	
≥ 50 years	651	29.8	70.2	
***Marital status***				0.007
Single/divorced/widowed	691	34.0	66.0	
Married	1502	28.4	71.6	
***Education***				0.35
Illiterate	984	31.0	69.0	
< 6 years	642	27.7	72.3	
≥ 6 years	578	30.4	69.6	
***Average family income (MAD)***				0.72
< 2000 MAD	1101	28.5	71.5	
2000 - 4999	541	28.8	71.2	
≥ 5000	188	31.4	68.6	
***Occupation***				0.18
Active/student	999	31.1	68.9	
Retired/unemployed/Housewife	1178	28.5	71.5	
***Housing***				0.002
Luxurious or modern	300	33.7	66.3	
New medina	340	26.5	73.5	
Old medina	423	23.6	76.4	
Poor housing or slums	1151	32.2	67.8	
***Tobacco consumption***				0.07
Current smoker	290	66.2	33.8	
Ex-smoker	185	65.4	34.6	
Never smoker	1632	71.3	28.7	
***Physical activity(≥ 30 min/day)***				0.34
Yes	1252	31.0	69.0	
No	780	29.0	71.0	
***Class BMI (Kg/m2)***				0.07
< 25	1241	30.1	69.9	
25 – 29.9	663	27.1	72.9	
≥ 30	300	34.3	65.7	

Sample of the adult Moroccan population, 2008. † Mediterranean Diet.

When entering all the variables associated with MeDi adherence at p < 0.20 in a multivariate logistic regression model (Table 4), the factors significantly and independently associated with lower MeDi adherence were rural origin, being single or divorced or widowed, luxurious or modern housing and being obese.

**Table 4:** Multivariate logistic regression analysis of factors associated with low MeDi adherence (score 1–4)

	**Adjusted OR**	**95% CI**	**p**
***Gender***			0.37
Women	0.88	0.67-1.16	
Men	1		
***Place of residence***			0.04
Rural	1.46	1.02-2.08	
Urban	1		
***Marital status***			<0.001
Married	0.68	0.55-0.84	
Single/Divorced/Widowed	1		
***Housing***			0.03
Luxurious or modern	1.66	1.18-2.33	0.004
New medina	1.15	0.82-1.63	0.42
Poor housing or slums	1.14	0.77-1.71	0.51
Old medina	1		
***Tobacco consumption***			0.14
Current smoker	1.23	0.91-1.67	0.18
Ex-smoker	1.38	0.97-1.97	0.08
Never smoker	1		
***BMI class (Kg/m2)***			0.006
≥ 30	1.56	1.16-2.11	0.003
25 – 29.9	0.96	0.76-1.20	0.70
<25	1		
***Occupation***			0.70
Active/student	1.05	0.82-1.34	
Retired/unemployed/Housewife	1		

Sample of adult Moroccan population without missing data, 2008. (N = 2183)

OR : Odds Ratio, CI : Confidence Interval.

## Discussion

The present study described the patterns of the MeDi in a sample of the adult Moroccan population and its association with socioeconomic and lifestyle characteristics. Our results show that correlates of MeDi adherence are different from those observed in other Mediterranean countries.

Indeed, contrarily to other studies [20-22], we found no association between age and MeDi adherence. In Mediterranean countries, especially those that are undergoing a nutritional transition, it is believed that younger people are more likely to adopt western dietary patterns. However, our study doesn’t support this hypothesis and this may be related to the beginning stage of the nutritional transition in Morocco. It may also be due to that female participants were younger and at the same time adhered more to MeDi. Therefore, younger subjects who were male (thus adhere less to MeDi) might not be well represented in the study population, leading to an overestimation of adherence in younger subjects. Nevertheless, our sample did not include children and adolescents under age 18, who are more prone to adopt this kind of dietary behavior.

We did not find significant differences in MeDi adherence according to either average family income or education. The same results were found in other Mediterranean countries [23,24]. Conversely, other studies showed a significant association between a healthier dietary pattern and both high education and income levels [25-27]. However, associations were found with housing categories in our study. In the new and old medina which still contains traditional houses, people are still keeping their traditional lifestyle which is maintained by availability of plants food, and their cheaper price. For people living in slums or poor housing, most of which are located in rural areas (results non shown), who are more likely living far away from markets, the low availability of food variety and the many years of drought that made them unable to grow foods in their gardens, could be an explanation for this departure from traditional MeDi.

Similarly, single, widowed and divorced persons tended to choose this westernized type of food more frequently than married ones, who seem more likely to maintain the traditional dietary habits in Morocco. This may be explained in part because married subjects eat more often regularly in family due to social obligations and share meals that are still prepared according to the traditional lifestyle.

Interestingly, higher BMI was positively associated with the lower MeDi adherence, which may be viewed as a consequence rather than a cause of the nutritional transition. The ATTICA study had already found a similar association in a Greek population [28,29]. Nevertheless, causality between obesity and the MeDi adherence cannot be drawn because of the cross-sectional design of the present study.

Many studies [16,30] showed that the presence of one or more cardiovascular risk factors such as hypertension, hypercholesterolemia and diabetes, is linked to a lower adherence to the simplified MeDi [29,31]. Unfortunately, information about these cardiovascular risk factors was not available in this study. But some previous Moroccan data have shown their high prevalence [32]. This may be partly explained by departure from traditional MeDi in Moroccan population.

The main limitation of this study could be due to self-reported food consumption. We used a FFQ to describe the dietary profile of the Moroccan population. Food consumption was not assessed with high accuracy and people might have overestimated the consumption of healthy nutrients and food items like vegetables, fruit or cereals, typical components of the MeDi and underestimated the consumption of unhealthy nutrients and food items like red meat and some fats. Moreover, as quantities of foods were not available, we could not neither adjust for total energy intake nor calculate the MeDi adherence score following the same steps as presented elsewhere [16,17] but only a simplified score. Furthermore, there was a lack of information regarding traditional CVD risk factors, such as hypertension, diabetes mellitus and hypercholesterolaemia in the study. This may explain some discordant results. Indeed, in poor housings or slums better adherence to a MeDi in terms of food choices may be counterbalanced by insufficient quantities of nutrients and low total energy intake. Another limitation is the cross-sectional design of our study which did not allow the identification of individuals who shifted from a traditional to a western diet. The cross-sectional design also does not permit to interpret as causal the direction of the observed association between lower MeDi adherence and obesity. Nevertheless, this study has several strengths. It was conducted in a large national population sample. Participants answered a detailed questionnaire and were especially motivated by trained interviewers to answer correctly the questions without causing misclassification. The results give for the first time a description of the adherence to a MeDi in the Moroccan population and its correlates taking into account the seasonality of consumption since the FFQ asked about dietary habits in the previous year. This information could prove useful, particularly when no other type of comparable data are available, in the formulation of dietary guidelines, and in public health initiatives involving nutrition policies and their implementation.

## Conclusion

The present study demonstrates that the MeDi is far from being a universal pattern in the Moroccan population. Adherence to a MeDi is often lost independently of age and education, especially in rural areas, among persons living alone and in those living in luxurious housing. These groups should be targeted in intervention strategies to stop the nutritional transition and to maintain the traditional MeDi pattern considered as the original diet in Morocco.

## Competing interests

P. Barberger-Gateau has received fees or support for conferences from Danone, Lesieur and Aprifel, and research grants from Lesieur and Danone.

## Author contributions

KE has contributed to conception and design, acquisition of data, analysis and interpretation of data and have been involved in drafting the manuscript; CN has contributed to conception and design, interpretation of data, have been involved in revising the manuscript critically and have given final approval of the version to be published. DR has been involved in drafting the manuscript CF has been involved in drafting the manuscript and have given final approval of the version to be published. MO has made substantial contributions to conception and design of data. AZ has contributed to conception and design of data. RB has contributed to conception and design of data. PBG has been involved in drafting the manuscript, revising it critically for important intellectual content and has given final approval of the version to be published. All authors read and approved the manuscript.

## Pre-publication history

The pre-publication history for this paper can be accessed here:

http://www.biomedcentral.com/1471-2458/12/345/prepub
